# Suture repair using loop technique in cases of acute complete acromioclavicular joint dislocation

**DOI:** 10.1007/s10195-011-0130-6

**Published:** 2011-02-17

**Authors:** Mohamed Taha El Shewy, Hatem El Azizi

**Affiliations:** 1 Orthopaedic Department, Cairo University, Cairo, Egypt; 2 Radiodiagnosis Department, Cairo University, Cairo, Egypt; 3 51(B) Demascus Street, Dokki, Cairo, Egypt

**Keywords:** Acromioclavicular, Dislocation, Repair

## Abstract

**Background:**

Acromioclavicular joint dissociation may not be a common injury, yet it may cause limitations in activity. Types IV, V, and VI dissociations need operative repair. In this study, a simple technique is advocated to reduce and maintain reduction of the acromioclavicular joint using no. 5 nonabsorbable suture material while the resutured coracoclavicular (CC) ligament heals.

**Methods and methods:**

Twenty-one patients (16 men and five women) with types IV and V acromioclavicular joint dissociation were studied. In all cases, acromioclavicular joint was reduced and reduction was maintained using no. 5 nonabsorbable suture material passed as a loop under the knuckle of the coracoid process and through a tunnel drilled through the lateral third of the clavicle. The CC ligament was then resutured.

**Results:**

Patients were followed up over a period of 6–9 years. At the final follow-up, all patients had returned to their preinjury level of activity, with significant improvement in the University of California Los Angeles (UCLA), American Shoulder and Elbow Surgeons Shoulder (ASES), and the Constant scores.

**Conclusions:**

This technique provided good results with no loss of reduction, except in a single case, during the long follow-up period. We could not prove that the good results are due to the healing of the CC ligament. However, patients were able to return to their daily activities and even contact sports without any noticeable deformity, feeling of weakness, pain, or limitation of range of motion (compared with the contralateral side). This technique does not involve the use of metallic implants, which require another surgery to remove them, the use of expensive synthetic graft, or a graft harvested from a distant donor site.

## Introduction

Acromioclavicular (AC) joint injuries may occur as a result of a direct force applied to the tip of the shoulder with the arm adducted or due to indirect trauma such as a fall on the outstretched hand. These injuries were classified according to Post [20] and later according to Rockwood et al. and Williams et al. [21, 28] into six types. Surgical treatment is advocated for types IV, V, and VI and in some cases type III where there is complete AC joint dissociation. This is due to the increased incidence of unsatisfactory results of conservative treatment of such cases [21]. Most of the surgical techniques described in literature involve the use of metallic devices to reduce and maintain reduction of the AC joint. Yet, the results, though good, are not totally satisfactory [1, 2, 8, 14, 18, 22, 24]. The metallic devices used are usually difficult to place and may be associated with complications. They require removal before the patient can return to normal activities [15]. Another option for surgical treatment is reconstruction of the coracoclavicular (CC) ligament [7, 10, 26].

This paper discusses the results of a simple, new procedure of surgical repair for acute complete AC joint dissociation (IV, V, and a few type III cases). The procedure involves the use of a loop of no. 5 nonabsorbable sutures passed under the base of the coracoid (knuckle) and through a tunnel drilled in the flat lateral end of the clavicle, with direct repair of the CC ligament. This maintains the AC joint in the reduced position until the CC ligament heals. This technique does not involve the use of metallic implants for fixation.

## Materials and methods

Twenty-one cases of acute AC joint dissociation were assessed in this prospective study. All patients had type IV or V AC joint injuries according to Rockwood classification. All surgeries were performed within 1 week of injury by the same surgeon between February 2000 and June 2003. All patients gave their written consent to undergo this type of repair. Also, we received the approval of our local ethical committee (Orthopedic Department, Cairo University) to undergo this study. There were 16 men and five women. The average age at the time of surgery was 31.8 (range 22.3–39.5) years. The duration between the time of injury and the date of surgery varied between 1 and 5 days, with an average of 2.14 days. The nondominant side was affected in six cases. Ten patients were injured while participating in sports (Table 1).

**Table 1:** Patient demography

Case .no	Age (years)	Sex	Dominant side	Sport	Duration between injury and surgery (days)	Mechanism of injury	Duration of follow-up (years)
1	37.2	♂	Dominant	Basketball	2	Hitting the ball-post^a^	9.5
2	23.4	♂	Dominant	No	2	Fall on shoulder tip	9.2
3	28.3	♂	Nondominant	Professional hurdles	3	Fall on shoulder tip^a^	9.0
6	36.2	♀	Dominant	No	1	Car accident	8.9
5	23.6	♂	Dominant	No	5	Fall on shoulder tip	8.8
6	38.4	♀	Nondominant	No	2	Fall down the stairs	8.5
7	29.8	♂	Dominant	Professional volleyball	1	Fall on shoulder tip^a^	8.3
8	35.7	♀	Dominant	No	2	Car accident	8.2
9	39.5	♂	Dominant	Squash	1	Hitting wall with shoulder tip^a^	8.0
10	26.5	♂	Dominant	Handball	2	Hitting goal-post^a^	7.8
11	36.2	♂	Dominant	Soccer	1	Fall on shoulder tip^a^	7.6
12	27.8	♂	Nondominant	No	2	Fall on shoulder tip	7.5
13	22.3	♀	Dominant	Volleyball	2	Fall on shoulder tip^a^	7.3
14	33.5	♂	Nondominant	No	3	Car accident	7.2
15	29.8	♂	Dominant	No	1	Fall down the stairs	7.0
16	34.7	♂	Nondominant	Soccer	3	Fall on shoulder tip^a^	6.9
17	37.5	♂	Dominant	No	2	Fall on shoulder tip	6.8
18	36.3	♂	Dominant	Professional handball	4	Fall on shoulder tip^a^	6.6
19	36.2	♂	Dominant	Basketball	1	Fall on shoulder tip^a^	6.4
20	29.4	♂	Nondominant	No	3	Fall on shoulder tip	6.2
21	25.7	♀	Dominant	No	2	Car accident	6.0

^a^ Participating in sports

Injuries were documented by preoperative plain X-rays of the affected shoulder in the anteroposterior (AP), lateral scapular, outlet, and axillary views. Similar plain X-rays were performed for the opposite shoulder. Patients were evaluated preoperatively using the University of California Los Angeles (UCLA), Constant, and American Shoulder and Elbow Surgeons Shoulder (ASES) scoring systems. This study was conducted according to the principles established in Helsinki and approved by the ethical committee of the orthopedic department of Cairo University.

### Operative technique

**Fig. 1:**
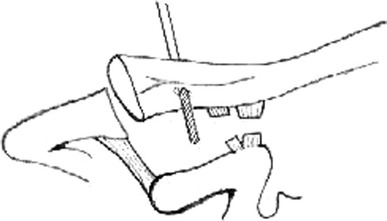
A 3.2-mm drill bit is used to make a tunnel in the middle of the flat surface of the distal end of the clavicle

Under general anesthesia, the patient was placed in the beach-chair position. A slightly curved 3- to 4-cm linear incision was performed over the AC joint and along the distal half of the clavicle. The deltoid muscle (with the attached periosteum) was elevated off the anterior edge of the distal third of the clavicle. In most cases, it was already stripped off the clavicle. The deltoid was slightly inferiorly retracted until the coracoid process was exposed. A 3.2-mm drill bit was used to make a tunnel in the middle of the flat surface of the distal end of the clavicle about 2.0 cm medial to AC joint (Fig. 1). A more lateral drill hole will cause forward pull of the distal clavicle, preventing anatomical reduction, which will interfere with the AC joint capsule resuturing.

**Fig. 2:**
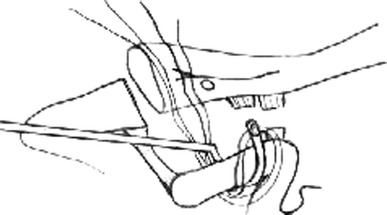
Curved aneurismal needle is used to pass two strands of the Ethibond sutures under the knuckle of the coracoid process

Two strands of no. 5 nonabsorbable Ethibond sutures were passed under the knuckle of the coracoid process using a curved aneurismal needle as a suture passer. It was then passed through the drill hole in the clavicle, in a figure of 8 manner (Fig. 2).

**Fig. 3:**
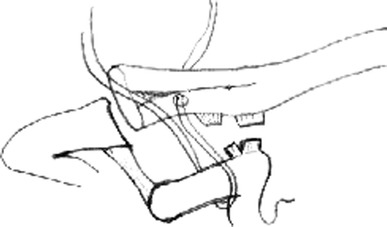
The two strands of the Ethibond sutures are passed through the drill hole in the lateral flat end of the clavicle in a figure of 8 fashion

The sutures are then passed under the knuckle of the coracoid and through the drill hole in the distal end of the clavicle (Fig. 3).

**Fig. 4:**
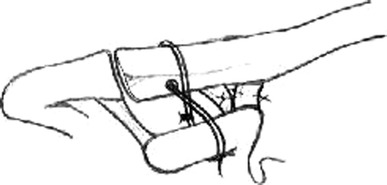
Acromioclavicular (AC) joint is reduced and the two ends of the suture material are tied securely to each other, forming a knot under the distal end of the clavicle. The torn coracoclavicular ligament is then repaired

Stay sutures were placed into both edges of the torn CC ligament using a no. 1 absorbable suture. In many cases, it was difficult to identify the conoid from the trapezoid parts. In such cases, both parts were sutured en masse. With the help of the assistant, the patient’s arm was pushed up while the distal clavicle was pushed down to achieve AC joint reduction. Then, while keeping the AC joint in the reduced position, the two ends of the no. 5 Ethibond suture were tied securely to each other, forming a knot under the distal end of the clavicle (between it and the coracoid) (Fig. 4).

**Fig. 5:**
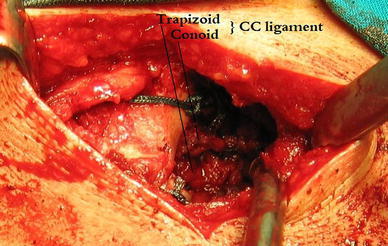
Conoid and trapezoid resutured

The edges of the CC ligament were then repaired by tying the previously placed stay sutures together (Fig. 5). If the CC ligament was stripped off the undersurface of the clavicale, it was reattached with two transosseous sutures using a 2.5-mm drill bit. This occurred in eight cases.

**Fig. 6:**
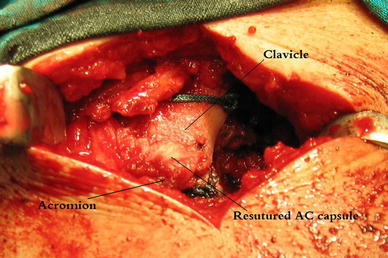
Resuturing acromioclavicular (AC) joint capsule

The ruptured AC joint capsule was then resutured. If it was avulsed off the clavicle (in seven cases), it was resutured to the clavicle by transosseous sutures using sharp bone-cutting needles (Fig. 6).

If (as in five of our cases) there was AP instability in the AC joint, another transosseous suture was placed in front of the AC joint to prevent anterior translation of the clavicle. The deltoid and the trapezius were then fixed back onto the clavicle using a no. 1 absorbable suture. The skin and subcutaneous tissues were closed in the usual manner.

#### Postoperative care

The patient was placed in an arm-pouch sling for 4–6 weeks postoperatively, during which light use of the hand was allowed. This aimed at supporting the elbow and arm to reduce the downward pull on the repair. Plain X-rays (AP and outlet views) were performed the first day postoperatively to document AC joint reduced position. Physiotherapy was started 6 weeks postoperatively with gradual return to normal daily activities. Sporting activities were allowed only when AC joint stability was established both clinically and radiologically. The latter was performed by doing stress views 6, 12, and 20 weeks postoperatively. After that, patients were followed every 6 months for the next 2 years and then on annual basis with special emphasis on recurrence of the deformity or osteoarthretic changes in the AC joint. Statistical analysis of the results was performed using the Wilcoxon signed-ranks test. At the final follow-up, all patients were reevaluated using UCLA, Constant, and ASES scores.

## Results

**Fig. 7:**
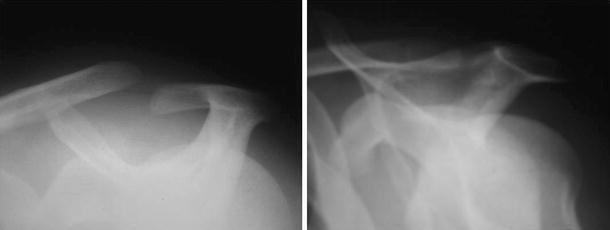
: Pre and postoperative outlet view

**Fig. 8:**
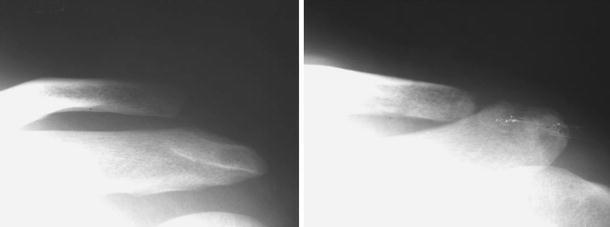
Pre and postoperative antroposterior view

**Fig. 9:**
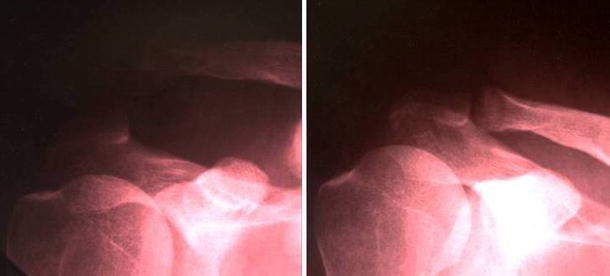
Pre and postoperative antroposterior view

Twenty-one consecutive patients underwent surgical repair for complete AC joint dissociation (types IV and V) injuries between February 2000 and June 2003. All patients underwent the previously described loop repair technique for stabilizing the AC joint. Preoperatively, all patients had aching shoulder pain, weakness, and deformity that interfered with their daily activities. They all complained of painful limitation of the active range of motion and weakness resisted forward elevation of the affected arm in contrast with the contralateral one (Figs. 7, 8, 9).

More than 50% superior displacement of the distal clavicle defined recurrence of deformity. Follow-up radiographs revealed maintenance of reduction in 20 of the 21 patients (95.24%). The one patient who sustained a recurrence was seen 6 weeks postoperatively without any deformity. He subsequently went back to playing squash, against medical advice, but his pain prevented him from continuing. Three months postoperatively, he presented with a recurrence of the deformity. He was followed clinically and did well by other objective outcomes measures. Other than this case, there were no intra- or postoperative complications (neurological or other types), except in one case. That patient suffered from superficial wound infection that led to wound gaping. This was successfully managed by prompt administration of systemic antibiotics for 7 days with repeated dressings. The wound finally healed by secondary intention. This patient complained about the appearance of the wound scar and sought the advice of a plastic surgeon. Although there was some anterior deltoid wasting in all patients during the first 4–6 weeks postoperatively, this almost completely disappeared in all cases by the 16th week (after 10 weeks of physiotherapy). At the final follow-up, none showed deltoid atrophy or detachment. During the long follow-up period, no patient developed clinical or roentgenographic evidence of AC arthritis. None showed distal clavicular osteolysis. Three patients developed ossification of the CC interval 3–5 years postoperatively. This was discovered in follow-up roentgenograms (all three did not participate in sports) but with no clinical effect. All patients were able to resume their daily activities. Seven who enjoyed preinjury recreational sports were able to return to sport at the same level. All three patients who were professional athletes were able to return to their preinjury sports performance level.

Results according to the UCLA score [6] showed a significant improvement in pain, function, active forward elevation, strength of forward flexion, and patient satisfaction. The mean total score improved from 52.8 to 95.0 at the final follow-up (Table 2).

**Table 2:** Pre and postoperative University of California Los Angeles (UCLA) score

Items	Preoperative mean ± SD	Postoperative mean ± SD	*P* value
Pain (10)	3.3 ± 1.0	9.1 ± 1.0	<0.001*
Function (10)	7.1 ± 1.0	9.6 ± 0.8	<0.001*
Active FE (5)	3.7 ± 0.5	4.9 ± 0.4	<0.001*
Strength of FF (5)	3.4 ± 0.5	4.9 ± 0.4	<0.001*
Overall patient satisfaction (5)	0.0 ± 0	4.8 ± 1.1	<0.001*
Total score (35)	18.5 ± 2.6	33.2 ± 2.9	<0.001*
Total score (100)	52.8 ± 7.5	95.0 ± 8.2	<0.001*

*SD* standard deviation, *FE* forward elevation,*FF* forward flexion

* Wilcoxon signed-rank test

The Constant score [5] showed a significant improvement in pain, activity level, arm positioning, strength of abduction, and range of motion (in all directions). The mean preoperative score was 63.3 and the mean final score was 97.8 (Table 3).

**Table 3:** Pre- and postoperative Constant score

	Preoperative mean ± SD	Postoperative mean ± SD	*P* value
Pain (15)	8.3 ± 2.4	14.8 ± 1.1	<0.001*
Activity level (10)	4.0 ± 0	9.7 ± 1.3	<0.001*
Arm positioning (10)	7.3 ± 1.0	9.7 ± 0.7	<0.001*
Strength of abduction 90º or highest level patient can achieve (Pounds) (25)	18.3 ± 3.4	24.6 ± 1.2	<0.001*
Range of motion			
Forward flexion (10)	7.3 ± 1.0	9.7 ± 0.7	<0.001*
Lateral elevation (10)	7.3 ± 1.0	9.9 ± 0.4	<0.001*
External rotation (10)	5.3 ± 1.0	9.7 ± 0.7	<0.001*
Internal rotation (10)	5.3 ± 1.0	9.7 ± 0.7	<0.001*
Total score (100)	63.3 ± 9.3	97.8 ± 6.2	<0.001*

*SD* Standard deviation

* Wilcoxon signed ranks test

The ASES score [11] showed a significant improvement in both pain and activity level, as well as the total score, which was 57.2 preoperatively and 95.0 at the final follow-up (Table 4).

**Table 4:** Pre- and postoperative American Shoulder and Elbow Surgeons Shoulder (ASES) score

	Preoperative mean ± SD	Postoperative mean ± SD	*P* value
Pain (50)	27.4 ± 4.1	46.7 ± 4.8	<0.001*
Activities of daily living (50)	29.8 ± 6.5	48.3 ± 5.5	<0.001*
Total score (100)	57.2 ± 8.3	95.0 ± 8.2	<0.001*

*SD* standard deviation

* Wilcoxon signed ranks test

## Discussion

Surgical management of AC joint injuries is indicated in types IV, V, and VI injuries and in some cases type III. Many surgical techniques described in literature involve the use of metallic implants for internal fixation. These implants include Kirschner wires [14], smooth or Knowles pins [8, 27], fully threaded pins [1], Arbeitsgemeinschaft fur osteosynthesefragen (AO) screws [18], and Balser hook–plate [22] with or without superior AC joint wiring, which acts as a tension band. Others place an AO screw [24] or a tension band [2] between the clavicle and the coracoid process. Others use modified Weaver–Dunn procedure with resuturing the released coracoacromial (CA) ligament to the clavicle via transosseous sutures in addition to placing a Bosworth CC screw that is removed after 8 weeks. In one study, there was a loss of reduction in two (11.8%) of 17 cases [19]. Many of these metallic implants are difficult to place and are accompanied by a high level of serious complications, such as pin migration [15]. They also need to be removed before the patient can return to normal activities, with the hazards of another surgery.

Other surgical techniques to stabilize the lateral clavicle include reconstruction of the CC ligament by transferring the AC ligament or using autograft or allograft tissues. Morrison and Lemos reported good and excellent results using a synthetic loop passed through drill holes in the base of the coracoid and the lateral third of the clavicle. They had good results in 12 of 14 cases [17]. Chen and coworkers achieved similar results, having overall satisfactory outcome in 41 of 48 (86%) patients [4]. Baumgarten and coworkers used an arthroscopcally assisted technique utilizing a semitendinosus allograft. This required a 3-cm incision and was technically tedious [3]. Others reconstructed the CC and AC ligaments for both acute and chronic cases using tendon grafts and supplemented the repair by temporary fixation (Kirschner wires) [13, 15, 29, 30]. Some surgeons resected the distal clavicle and reinserted the CA ligament intramedullary (docking technique). They used it mainly in chronic cases, achieving good results with loss of reduction in one (6%) of 16 cases [16]. A technique was described involving transfer of the lateral half of the conjoined tendon to the distal clavicle in a proximally based fashion with loss of reduction in 11% of cases. This technique required additional CC fixation that later needed removal [9]. Other surgeons dissected the CC ligament and refixed it to the undersurface of the clavicle using transosseous sutures or anchors. This was done by open technique, with loss of reduction in five (17.2%) of 29 patients [23]. It was also done via an all-arthroscopic procedure [12, 19]. Tienen and associates transferred the CA ligament to the clavicle and fixed it by absorbable, braided suture cord. There was failure to achieve anatomical reduction in three (14.4%) of 21 cases [25]. All these techniques aim at providing a stable AC joint without the need for metal fixation. However, some techniques required additional fixation to avoid loss of reduction.

The loop technique described here is a novel technique that provides a simple yet stable method for reducing and maintaining reduction in cases of AC joint disruption. In addition, it does not involve the use of metal implants, which require later removal. Also, it does not involve expensive synthetic grafts. The incision used in this technique is small (3–4 cm). The duration of postoperative immobilization is relatively short. One of the difficulties of this technique is the very small size of the CC ligament (about 10–13 mm). This makes its handling quite difficult. In most cases, the CC ligament edges are resutured en masse after failure to identify the conoid from the trapezoid.

A drawback of this study is that we could not attribute the good results achieved to the healing of the CC ligament or to the suture material used to maintain the reduction, or to both. Only a magnetic resonance imaging (MRI) study could clarify this issue, but this could not be done, as it was extremely difficult to convince a patient after recovery to perform the expensive MRI study.

In conclusion, we believe this technique should be used only in acute cases. Our results show a high incidence of good outcome, with loss of reduction in a single case only, during the long follow-up period. We could not prove that the good results are due to CC ligament healing. The patients were able to return to their daily activities and even to contact sports without any noticeable deformity, feeling of weakness, pain, or limitation of range of motion (compared with the contralateral side). The technique does not involve the use of metallic implants, which require a second surgery to remove them, or the use of expensive synthetic graft or a graft harvested from a distant donor site.
